# Developing a CPD model for Eswatini—a participatory action research study

**DOI:** 10.1186/s12909-023-04016-7

**Published:** 2023-01-20

**Authors:** Rodney Hudson Magwenya, Andrew Ross

**Affiliations:** 1 Mankayane Gvt Hospital, Mankayane, M206 Eswatini Swaziland; 2 Department Family Medicine, University of KwaZulu Natal, Private Bag 7 Congella 4013, Durban, South Africa

**Keywords:** Participatory action research, Continuing medical education, Continuing professional development, Entrustable Professional Activities

## Abstract

**Background:**

Continuing professional development (CPD) is a key aspect to fulfil a commitment to lifelong learning for professionals registered with the Medical and Dental Council, the intention being to promote the health of patients and develop clinical expertise. The absence of formal CPD requirements for practitioners in Eswatini has resulted in a move to introduce an accredited system.

**Methods:**

The qualitative study followed a participatory action research (PAR) methodology using a cooperative inquiry group of 10 medical practitioners in Eswatini to investigate how the current CPD program could be improved and formalised. PAR entailed four stages; observation, reflection, planning and action, using a semi-structured format to explore the areas of concern.

**Results:**

Reflecting on the current situation resulted in three ways to improve CPD being identified: (1) adopt a formal, compulsory CPD model; (2) recognise achievements by practitioners who endeavour to improve their skills/knowledge through Entrustable Professional Activities, and (3) ensure that CPD is relevant to the workplace by using Quality-Improvement CPD (QI-CPD) and reflective diaries. These would be done by involving local practitioners, using adult learning principles and ensuring continuous evaluation and improvement of the CPD model.

**Conclusions:**

There was general agreement on the need for a formalised CPD system to improve skill levels and provide an open platform to enhance patient care in a resource constrained setting. The findings provided information that can be used to plan and action its implementation through engagement with the country’s doctors in various forums and through ongoing research.

## Background

Continuing professional development (CPD) is a key aspect in the fulfilment of a commitment to lifelong learning for health care professionals, the intention being promote the health of the patients and develop clinical expertise [1, 2]. Although there are some informal CPD activities, the Medical and Dental Council (EMDC) in the Kingdom of Eswatini has identified the need to develop a formal / compulsory CPD model.

Eswatini is a small southern African country with a population of around 1.172 million people [3]. It has around 336 registered general medical practitioners, the majority of whom are working in the public service in one any one of the six government hospitals, two mission hospitals or five health centers [4, 5]. There is a growing private sector, but it caters for a minority of the population. There is currently no medical school in Eswatini, and all doctors practicing in the country trained in many different countries (including but not limited to recognised medical schools in South Africa, Taiwan, Russia and China). In 2018 an internship programme was established for newly qualified doctors returning from their studies and all doctors wishing to practice in Eswatini must pass registration examinations [5]. However, there is a significant adjustment and adaptation that is required before newly registered doctors can practice optimally, due to the differences between where they studied and where they will practice. Current CPD programmes which should help bridge this gap may not exactly prepare them for this.

The available CPD opportunities are mainly hospital based in-service programmes run by local CPD committees, which cater for all healthcare workers in a facility. They have widely varying effectiveness and rely on the willingness of staff members to participate as there is no obligation to attend. A few other opportunities are available through the Ministry of Health and its partners; these are mainly targeted programmes (such as clinical updates for specific conditions under specific ministry projects). As of May 2022, there are no enforced CPD targets, although all those registered with the medical council (health care practitioners) are encouraged to participate. Practitioners are encouraged to report the CPD programmes they attended over a year when they submit their annual practice license renewals, though currently this is not a precondition for renewal [5].

In a majority of CPD programmes (Eswatini included) the target audience are often excluded from policy and other decision-making processes [6–8]. Faghihi et al. (2017) contend that when there is a disconnect between the CPD offerings and the needs of practitioners, the programmes are likely to be impractical and not well received [8]. Determining the needs of practitioners is an important step in developing effective CPD programmes [9]. Continuing professional education is at its fundamental level adult education [10]. As such it should be underpinned by adult education theories and principles. Andragogy (adult leaning theory) refers to the methods used by adults to learn, their learning process being different to children, and among themselves in terms of needs and styles [10]. It was proposed by Knowles who made five assumptions about the way adults learn [11]. These include self-concept (self-directed learning); adult learner experience (drawing from previous experience in order to learn new things); readiness to learn (easier to learn when there is a reason to acquire new knowledge or skill); orientation of learning (learn things that are applicable to their practice) and motivation to learn (want to learn for intrinsic factors, e.g., to improve self-esteem, advance their careers) [11]. Furthermore, Knowles added four principles to be applied to adult learning. Firstly, that adults need to be involved in the planning and evaluation of their learning activities, this has important bearing when most CPD activities are concerned as they tend to be provider-driven than learner-driven. Secondly, experience including any mistakes made provide an important basis for learning. Thirdly, adults prefer those learning activities that are most relevant to them and impact on their work. That forms the basis of needs assessment in CPD. Fourthly, adult learning should be problem centred rather than merely content-oriented [11].

Participatory action research (PAR) is a research methodology in the emancipatory –critical paradigm that combines equal involvement of research participants and researchers in the research process (participatory research), using the findings to address issues affecting a particular group (action research) [12–14]. Action research consists of multiple cycles each with three main stages, namely inquiry, action and reflection [15]. Participants are regarded as experts due to their lived experiences, and their participation in the collaborative work with the researcher ensures relevance of what is being studied, which in turn produces findings that present some possible solutions to practical problems [12, 14], the collaborative relationship forming the basis of the cooperative inquiry group (CIG) [14]. In a CIG, individuals, including the researcher, come together to explore matters of interest as ‘co-researchers’, with the aim of transforming their (environment) practice [14, 16]. PAR can thus promote changes desired by a group in terms of policy development and practice, in this case around CPD. Personal dissatisfaction with some CPD activities that were on offer led to a need to examine if there were ways in which the professionals could have a better CPD experience while also trying to improve patient care. There have been proposed methods of ensuring PAR meets the required quality and rigor following criticism of the original description by Kemmis and McTaggart [15] that was thought to lack well defined criteria to consistency between researchers’ philosophy and methodology [17]. Proposed criteria to ensure quality include alignment of group members with purpose, democratic and collaborative group dynamics and facilitation, development of reflexivity, commitment to practical action and detailed documentation of the process [14, 17, 18].

This paper seeks to explore how CPD can be improved for medical doctors practicing in Eswatini to enable policy makers and other stakeholders to know what doctors think should be included in the CPD model being developed.

## Methods

The qualitative study followed a participatory action research methodology using a cooperative inquiry group process. The participants were recruited from two public sector hospitals in Eswatini, and the data collection process occurred from December 2021 – February 2022.

### Selection of participants

Convenience sampling was used to identify suitable participants, with 10 – 15 being required for the group discussions. Initially, all 12 doctors at the hospital where the author is based were asked to participate, with an additional five at the nearest facility being invited to join the process to achieve the desired number. The participants involved were limited to general medical practitioners working at the two Government Hospitals in Eswatini to create a relatively homogenous group. Presentations were made to potential participants at locations and times convenient for them, and interested individuals were asked to sign an informed consent until the required number was reached. Of the 17 doctors who were approached, 10 agreed to take part.

### Data collection methods

The initial aim of the study was to develop a CPD model for Eswatini, which was discussed within the group to establish its suitability for the purpose of the process, as this informed the direction of the discussion. Two data collection methods were used: these being group discussions and research diaries. Participants were encouraged to keep a personal diary of their CPD and participation experiences to enable different perspectives to be documented, compared with and used in the reflective processes of the cycle [12, 14].

### The cooperative inquiry group process

The participants received initial basic training on the PAR methodology (from the author), CIG and reflectivity [14, 19]. This was an important stage that allowed the author to relinquish power (over the participant and the process) to the group, which enabled everyone to be aware of their role and contribute fully to the process. The aim was discussed and after the group deliberations, the research question ‘How to improve CPD in Eswatini’ was adopted.

**Fig. 1:**
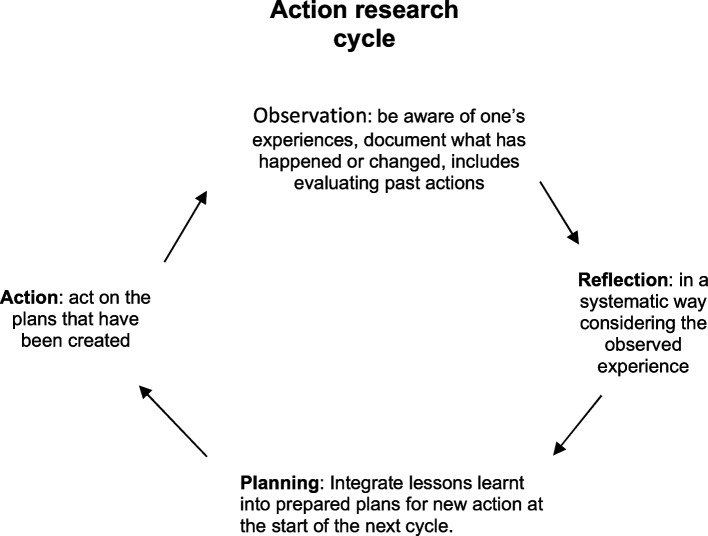
The four components of the action research cycle [12, 20, 21]

The study CIG process entailed four components of the action research cycle, these being observation, reflection, planning and action (Fig. 1). Attention was also given to the group process to ensure commitment and maximum participation. Participants were involved at all stages of the project [12, 14]. For each major stage of the study the group observed their experiences, and then reviewed the experience considering what was undesirable in their observed experience or what changes they wanted. The plans were put into action and later the cycle repeated only this time the observation looked at the changes done in the previous cycle. Fig. 1 is a simplified representation of the stages of the process which is at times depicted a spiral [12, 20, 21]. The number of times the cycle was repeated was different for each issue deliberated upon, with an average of two to three for each.

### Data collection, analysis and reaching a consensus

The discussions were held in English, recorded, transcribed verbatim, with participants receiving a summary of the meetings for review and corrections.

Consensus was built mainly using group discussion, and in the few issues were this was a challenge, a nominal group technique was employed, in which participants wrote down their ideas, then selected what they considered to be the best idea and presented it [14, 16]. After all the ideas were compiled, they were discussed in turn, wording of an idea was only changed if its originator agreed, and duplicates were struck off, while other ideas were removed by unanimous agreement. Multi-voting was used to prioritise the recorded ideas [14, 16]. A total of 12 meetings were planned and conducted, the numbers varying for each, with seven practitioners being able to attend all meetings.

### Ensuring the quality of the process

To ensure the quality of the PAR process, several steps were taken, in line with the criteria proposed for the appraisal of cooperative inquiry. Firstly, in terms of ownership of the inquiry process by members was ensured by training them on the research methodology to prevent the author from dominating the inquiry. Secondly, there was an emphasis on developing reflectivity in all aspects of the inquiry, which was done by sensitizing the members to the importance of reflectivity and encouraging open mindedness and critical thinking [19]. Thirdly, members were encouraged to share their thoughts freely without fear of judgment, and democratic and collaborative group facilitation was followed as much as possible through the inquiry. Fourthly, the process was documented in as much detail as possible, where individual experiences, group processes, reflections and final consensus were documented, and individuals had an opportunity to make relevant corrections and additions where they felt their ideas were not clearly addressed.

## Results

### Participants

The 10 participants consisted of four female and six male general practitioners, with between two and 15 years of post-qualification experience, the youngest being 27 and oldest 49 years old.

### CIG Process

In the CIG we examined what it meant to have a new CPD model and its desired characteristics. After deliberations, consensus was reached that the research topic should be ‘how to improve CPD’ rather than ‘developing a new CPD programme’. We further explored what improving CPD meant for us and decided that over the next six months we would:

- 1. Make observations about the current CPD processes and possible options to improve the programme.
- 2. Reflect on the current CPD activities and how we felt about it, whether it met our expectations and was making any meaningful contribution to our practice.
- 3. Plan how we could improve CPD to enable our experiences to meet our expectations.
- 4. At the end of six months we agreed on three main areas to act on, through a nominal group technique. The first was a structured CPD model, the second was that the CPD program needed to recognise of the efforts made by practitioners to develop themselves, and the third was that CPD needed to be relevant to local practice area.

#### A structured CPD model (policy development)

Using the Action Research Cycle there was an observation that there was a lack of a clear CPD policy in eSwatini. Upon reflection we developed consensus that there needed to be a clear policy and structure that could be implemented and subject to continuous evaluation. The following points were agreed upon as being important for CPD in Eswatini. The further action agreed upon was to make available the summary of the deliberations to the medical council for consideration when they were drafting the CPD policy.

1. CPD was a necessary part of the profession, and the new model should be made mandatory.
2. The model should also follow Association of Medical Councils of Africa (AMCOA) CPD protocol which the country has already committed to implementing [22]. The principles of which include participation by all practitioners, mutual recognition of CPD done in member states, monitoring of compliance and penalties for not reaching CPD targets, aligning and developing policy frameworks to comply with the protocol [22].
3. Annual targets were important. There were two main contributions regarding the mandatory nature of CPD, on one hand there was a group that felt that a stepwise implementation would be most ideal, while another felt that there should be mandatory CPD targets from the onset of this new model. Given that local CPD opportunities are not widely available; the group calling for stepwise CPD felt it was important that CPD point targets only be made enforceable once there were enough CPD opportunities for all practitioners in the country. The group calling for enforceable targets from the onset countered that there were already enough relevant CPD opportunities available online from various neighbouring and other countries. They added that the only issue was to incorporate these online CPD activities in a relevant framework to allow them to be recognisable for CPD credit. The latter became the consensus conclusion.
4. The Medical Council needs to establish a CPD committee to formally draw up clear CPD guidelines to be given to all practitioners on registration and to make them available on the council’s website
5. The composition of this committee must include senior practitioners (both specialists and general practitioners) in the country.
6. CPD activities should follow established adult learning theories, be based at the workplace to make them relevant to practitioners and be oriented to patient outcomes.
7. The CPD model should also be guided by varied sources of performance and outcome data, with a view of using these to improve on itself  [4, 23, 24].
8. CPD should be viewed as a collective responsibility of all stakeholders in the health system and fully integrated to it.
9. Recognition of achievement should be promoted
10. Impact on practice should be one of the main goals of CPD

#### Recognition of achievements

There was an observation that in the current system there was no formal recognition of the effort taken by practitioners to stay up to date or even to improve their clinical skills. In our reflection, most practitioners felt discouraged by this. Furthermore, there was agreement that if efforts were recognised these should be tied to a salary notching scale and could also lead to better satisfaction amongst practitioners and attendance at CPD activities.

*‘It is disheartening to know that no matter how much I improve myself through various CPD activities I take part in, I will still not get any formal recognition. For example, some of us have done some courses in HIV management and have not had any acknowledgement from our employers or medical council.’ (TD, 25yrs Female).*

*‘When I finished my training, I had to learn some skills as a medical officer. These included doing some surgical procedures like caesarean sections. However, there was no formal way to acknowledge that I had successfully acquired these skills. This is very disappointing, because when I switch hospitals, they only have my word that I’m able to do certain procedures.’ (MM, 44yrs Male)*

*‘It would be nice to have a way to formally recognize all the skills that I have made an effort to acquire during my practice’ (RM, 40yrs Male)*

**Fig. 2:**
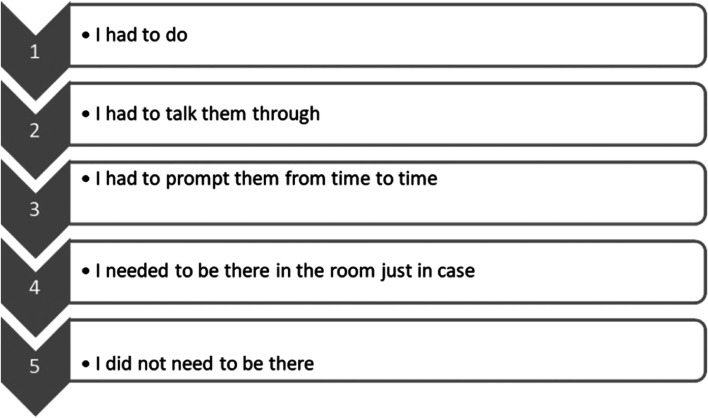
Ottawa entrustment scale [29]

In our planning we reviewed literature on CPD and discussed attending courses / hospital based CPD, journal clubs, reading of journals and completing questionnaires, attending accredited courses and postgraduate training, all of which play an important role in CPD. However, the group decided to focus on Entrustable Professional Activities (EPA’s) because not only do they provide a way to formally recognize training that occurs at the workplace, but they can also be made to be a part of CPD [25–27]. Entrustable Professional Activities (EPAs) were initially proposed by the Association of American Medical Colleges (AAMC), for use in medical education to provide a practical approach when assessing resident competence in carrying out defined tasks [26, 27]. They are units of professional practice that use observable work descriptors and enable the formalisation and justification of trainee entrustment measurement [26].The use of EPAs allows individuals to have proof of leaning a new skill, and if they are standardised nationally, would ensure that when people are being asked to do certain tasks their level of competence in those tasks has been formally assessed. For recognition of important skills learnt there was general agreement that EPAs would meet that goal. Due to the complexity in developing EPAs, we used a simplified system that looked at a few examples of possible EPAs basing this on the available literature (see Tables 1 and 2) [25–28]. We discussed some entrustment decision scales for use with EPAs, such as the Ottawa scales (both original and modified), and the Chen entrustment scale (original and modified) [29]. Through a consensus the original Ottawa scale (see Fig. 2) was chosen as it was simple, used in several studies and applicable to post medical school EPAs (unlike the modified Ottawa scale) [29]. It shows how an individual can progress along the 5 levels on entrustment (see Fig. 2 below).

**Table 1:** Some identified skills for EPAs

General	Medical	Paediatrics	Surgical	Obstetrics and gynaecology
History taking	Lumbar puncture*	Assessment of the newborn	Preoperative assessment	Caesarean section
Prescribing*	Pleural tap	Nasogastric insertion	Insertion of an intrathoracic chest drainage tube*	Evacuation of retained products of conception*
Obtaining informed consent	Ascetic tap	Intraosseous cannula insertion*	Incision and drainage of an abscess	Bartholin abscess management

*EPAs selected for further development

**Table 2:** Example of an EPA for lumbar puncture [30]

Key competencies	Behaviour requiring corrective action	Developing behaviours		Expected behaviour for an Entrustable practitioner
Technical skills	Lacks required Skills, Fails to follow sterile technique	Variably Applied, completes procedure Unreliably, Uses universal precautions and aseptic technique inconsistently	Approaches procedures as mechanical tasks and often initiated after request of others, Struggles in adapting approach when required	Makes necessary preparation before performance of procedure, Correctly performs procedure on many occasions over time. Makes consistent use of both aseptic technique and universal precautions
Knowledge	lack of awareness of knowledge gaps	Does not understand key Issues like indications, contraindications, risks, benefits, has limited, knowledge of procedural complications or prevention of these	Describes most of these key Issues Has knowledge of Common complications but struggles to manage them	Has adequate knowledge of essential anatomy, physiology, indications, contraindications, risks, benefits, and alternatives for the procedure Knows and can take steps to prevent and manage complications
Communication	Does not communicate effectively- Uses inaccurate Language, presents distorted Information, Disregards patient’s and family’s wishes Fails to obtain appropriate consent before performing a procedure	Uses jargon or other ineffective communication techniques unable to read emotional response from the patient, fails to engage patient in shared decision making	Conversations are respectful and mostly free of jargon and considers patient’s wishes, When focused on the procedure may have difficulties recognising emotional response from the patient	Demonstrates patient-centred behaviour such as avoiding jargon, enabling shared decision making, and regards patient’s emotional response), obtains appropriate informed consent
confidence	Overconfident takes actions that could endanger patients or others	Displays a lack of confidence that increases patient’s stress or discomfort, or overconfidence that erodes patient’s trust when practitioner struggles to perform the procedure, Accepts help when offered	Asks for help with complications	Seeks timely help Has confidence commensurate with level of knowledge and skill that puts patients and families at ease

In our action stage we looked at identification of EPAs. Following a week-long job task analysis some units of work which could be developed into EPAs were identified. These needed to be activities that could be easily observed, measured and suitable for making an entrustment decision. The table (Table 1) below shows some of the identified tasks.

A few EPAs were chosen by consensus to develop further for exploration in this project. These are marked with an asterisk. Table 2 shows an example of an EPA, though this is not the assessment tool it is adaptable to most procedures.

The development of EPAs for our ‘pilot’ was largely welcomed by participants. A fair number (6 out of 10) were hopeful that if introduced and properly standardized, EPAs could enable recognition of and improvement in skills acquired.

*‘I think EPAs provide a structured way of not only learning skills through description of what’s expected, but also allow one to demonstrate their competence in an objective manner. I really am hopeful that these may ease my concerns of recognition of work done by practitioners in upgrading themselves or learning new skills’ (MM, 44yrs Male)*

*‘I enjoyed the use of EPAs during the pilot. Even though I had done lumbar punctures many times when I was being assessed I found that I had to work harder at communicating with the patient than I have previously done and the requirement from the EPA ensured that I did so. If we can develop them for our routine tasks, we may be able to also improve our standard of care.’ (BT, 28yrs Male)*

‘EPAs initially were sounding very daunting to me, but once I was able to assist in developing the one for prescribing, I realized how useful they could be’ (MN, 27yrs Male)

A minority (2 out of the 10) still felt apprehensive and was unsure if these EPAS would be useful for them.

*‘I am still not convinced that EPAs will have the desired impact here, they seem more like what can be done at teaching hospitals. I think they may add more work for overburdened clinicians’ (TG 38yrs female).*

However, there was consensus that EPAs should be explored as a possible way of recognizing competence / achievements.

#### Making CPD relevant to the workplace- Quality improvement CPD (QI-CPD)

There was a general observation that CPD needed to assist in improving the quality of practice, with the participants reflecting that activities are often not relevant to their practice area.

*‘I strongly feel that CPD has to help improve me so that I can help my patients. This isn’t the case with most CPD activities I have done in the past’ (RM, 40yrs Male)*

*‘I wish that CPD could be helping address important issues I struggle with at my *facility* rather than just being another useless academic exercise.’ (BT, 28yrs Male)*

In planning we looked at approaches to CPD that are workplace based and have been associated with improved patient outcomes [24, 31–33]. Several activities were considered including the development of a learning plan, quality improvement projects, peer review of patient charts, the use of a diary and reflective practice. Quality improvement CPD (QI-CPD) and the use of a diary (see following section) and reflection were chosen by consensus as ways to ensure that learning is relevant to the workplace and it had a clear well-developed process.

In the action phase we identified by brainstorming the needs of practitioners based on their peculiar work environment and tailored to patient/clinical outcomes. Then, short term and long-term educational initiatives were planned to meet the identified needs. Lastly every practitioner engaged in a quality improvement project, guided by local objective outcomes and performance data. It was not possible to compare practice to benchmarks or peers due to the unavailability of relevant data [23]. Quality improvement was done through adaptation of the Plan–Do-study-Act cycles [34]. Two of the projects that were done as part of the QI initiative are summarised in Tables 3 and 4.

**Table 3:** Summary of the quality improvement project 'Diabetic clinic'

QI-CPD description	Identified need (gaps)	Goals	Educational initiatives to address gaps	Objective outcomes
Diabetic clinic	At one facility there was no clearly defined diabetic clinic, yet the hospital served may diabetic patients apparently there had been a diabetic clinic some years back but when the medical officer responsible for it left it was not continued by the new officers	1. To formally start a diabetic clinic 2. Have clear standards of care expected for each diabetic patient attending the clinic, such as scheduling of routine tests like renal function tests and referrals for eye examinations 3. ensure checklists used for each visit to screen for complications 4. use above tools as part of the data collection 5. evaluate the project	1. Sensitisation of the care team about the diabetic clinic. This included nurses, laboratory technicians, dietician/nutritionists, pharmacy, medical practitioners, physiotherapists, and counsellors 2. short interactive case discussions 3. Some members attended external courses on clinical care of patients with diabetes	1. A formal diabetes register was created 2. Record keeping for patients was improved, was done using tools that had been long available but seldom used 3. Was better compliance with national treatment guidelines and standards of care 4. Was improved follow-up tracking and earlier referrals for those with complications

**Table 4:** Summary of the quality improvement project 'prescription audit'

Stage	QI-CPD description
Identified needs	1. Complaints from the pharmacy department on the issues of polypharmacy and inappropriate prescriptions 2. No formal prescription audit had been done before practitioners in Eswatini trained at many different countries, each with own standards of care. Standard treatment guidelines are not readily used where available
Goals	1. ensure that prescriptions met appropriate general standards (legibility, identifiable prescriber, generic drug name use) 2. comply with Standard treatment guidelines (STGs) 3. review medications and treatment for chronic diseases 4. interaction checking 5. instructions and warnings to patients
Educational initiatives	1. sensitization of all prescribers and pharmacy about the audit and its scope 2. short interactive presentations on prescribing standards (adapted from the World Health Organisations ‘Guide to good prescribing’) [35] 3. promoting use of the STGs and some short interactive case discussions on management of common cases in the facility
Objective outcomes	1. audit findings (from the audit of prescription scripts) were presented in an open forum to prescribers 2. standard operating procedures for non-compliant prescriptions were developed 3. improvement in documentation including: legibility, use of generic drug names, documenting instructions/warnings to patients, 4. reduction in the number of drugs per prescription when comparing before and after audit

### Making CPD relevant to the workplace-reflective diary

In the observation stage, we noted that practitioners rarely reflected formally on cases that they were involved in managing. Part of the reason was lack of training on reflectivity. We looked at how reflection can become a regular part of practice and how CPD can play a role in its formal adoption by practitioners. A consensus was reached after nominal group method that a reflective diary is one way to encourage this. The main objections were related to the time required to complete the reflective diary.

Of the 10 practitioners, seven completed their reflective diaries. The first step required them to choose a specific case which had an impact on their practice or on them personally. This step was the easiest for all that responded.

*“I had so many cases I could think of that have been pivotal in my career. So, choosing one was not too difficult” (TG, 38yrs female).*

The second step was to share details about their thoughts, feelings, behaviours and actions. For some, detailing how a particular case made them feel was a new experience.

*“I found it challenging to write down my feelings about cases. I guess we are taught to dissociate our personal feelings about cases to keep us as objective as possible. However, mentioning my thoughts helped me think more deeply about my experiences and patient encounters.” (BT, 28yrs Male)*

The third step involved formulating learning needs from their experiences. Most practitioners were able to link several needs from each case they discussed.

*“From my case, I recognized that I needed to know about managing electrolyte imbalance. I also needed to know how to deal with stigma around drug resistant Tuberculosis among the different cadres”* (TG 38yrs female).

The fourth step involved listing the strengths and weaknesses of behaviour or action. Many participants felt this was an eye-opener for them.

*“Looking at the strengths and weaknesses of my behaviour or actions allowed me to critically assess my actions and forced me to look at them in an unbiased way”* (TD, 25yrs Female)

*‘In my daily practice I rarely get time to look back at cases due to the sheer number of patients I must see. However, I now appreciate how important it is for me to reflect on my actions so that I can improve and where necessary make better decisions next time (TG,38yrs Female)*

The fifth step was to explore other strategies and approaches. For many this was useful, and they felt it prepared them for the next time they met with similar cases.

*“In my case I would have preferred more guidance from specialists for some of the complicated interventions especially for paediatric patients. However, the favourable outcome for this particular patient also prepared me to manage similar cases in the future” (TG, 38yrs female).*

The last step involved some conclusion of learning points and making smart specific action plans for change of practice. Most participants came up with clear SMART (specific, measurable, achievable, relevant, and time bound) action plans.

*“For my smart action plan, I will be discussing with the paediatricians at my referral hospitals for them to provide us with guidance on managing electrolyte imbalance in paediatrics. Such a guideline will be the measurable outcome together with outcomes once it’s implemented. I am hopeful that this can be achieved in 2 months” (MN, 27yrs Male)*

While allocation of CPD points may be deemed to be an objective way to measure whether one has participated in CPD activities it may not necessarily be the most effective way especially when it comes to reflective diaries. The complexities and differences between cases would make this difficult. There was therefore consensus among participants that if diaries were to be used, then certain minimum standards could be put in place. These could involve for example prescribing a minimum of two reflective cases per CPD cycle. A CPD cycle (which can be 2 years for example), being the time to accumulate a recommended minimum number of points/credits. There was also a recommendation that this reflective diary be made into an electronic or web-based application for ease of use.

‘Diaries are a significant strain in our busy work schedules but if they are to be implemented having them on as app may make it easier for us’ (BT, 28yrs Male)

### Evaluation

One of the greatest challenges faced was capacitating practitioners about PAR which was a new concept for them. It did take more time than had been anticipated and there still were challenges as some had to be encouraged repeatedly to voice their ideas and thoughts. For the authors it was also challenging to try and be part of the process rather than be the one driving it. However, the level of teamwork and engagement with other participants made the experience worthwhile. Choosing a different team member to lead each of the different sessions helped members get more involved in the process.

Many of the participants where initially apprehensive about the PAR but were able to realise its potential in addressing some concerns they had around current CPD activities in Eswatini.

*‘The work before us was scary, looked insurmountable, but the process we followed was able to help us make significant progress in getting our thoughts known” (RM, 40yrs Male)*

For a lot of others, they gained some skills which they felt would help them in their practice.

‘I had so much to learn, so many new words and ways of thinking’ (MN, 27yrs Male)

‘If anything was achieved in this project it was to open my eyes to look at myself and see the many ways I could improve’ (LN, 36yrs Female).

The majority also felt that a lot of work was needed to investigate practicalities of these interventions we had suggested above.

*‘This project is just the beginning. We hope for more opportunities to further explore how we can improve ourselves as in doing so our patients will benefit the most’ (MM, 44yrs male)*

## Discussion

Participatory action research represents a significant change in the way that healthcare research can be done. However, it is a useful methodology to explore our current topic. To a large extent the process was effective, mainly due to the enthusiasm of participants and their willingness to participate. The greatest challenge was to equip all participants with enough knowledge about PAR so that the group discussions would not be dominated by a few individuals. It was also difficult for participants to use the diary (both in terms of time constraints and it being a new concept for some), and the reflective practice was relatively new for almost all of us. We had hoped for answers to our questions but ultimately realized that there were many things that still needed further investigation using perhaps other methodologies as well. However, there was clarity on several issues.

### A structured CPD model (policy development)

CPD is an important part of equipping practitioners with the skills and knowledge to meet the challenges they encounter in their daily practice. Literature highlights that the quality and quantity of the CPD activities undertaken also has a correlation with the quality of professional practice [36, 37]. There are many models of CPD that can be developed and implemented in Eswatini, and any such model needs to be of appropriate quality for it to have meaningful impact. The chosen model needs to involve practitioners with some understanding of the local practice environment and be grounded in the workplace, while also being integrated into the health system [38]. The CPD model should also be based on adult learning principles and be oriented to patient outcomes [38]. This can be achieved by putting in place a well drafted CPD policy which considers all the contributions of stakeholders.

### Recognition of achievements (EPAs as an example)

Entrustable professional activities (EPAs) are specific units of professional practice which, can be entrusted to an adequately competent individual (professional). They are a relevant consideration for inclusion in the CPD model as they can be made to be appropriate to the duties of practitioners and they can potentially be useful in improving patient outcomes by ensuring a standard level of performing certain task is maintained [39]. They also provide documented evidence of competence to perform a specific activity which has been shown to be a motivation factor for engaging in CPD [4, 25]. Indeed, EPAs should be suitable for credentialing, that is establishing the level of skill for a professional and assessing their legitimacy [26]. In Eswatini they can be developed nationally by specialists working with other experienced medical practitioners for each core competency required of a practicing medical officer [25]. To simplify the documentation and entrustment-decision making of developed EPAs, they can be added to an electronic portfolio [27]. In each facility, specialists or other experienced medical practitioners with sufficient training in each EPA can be responsible for assessments of competence using a scale like the one described in Table 2 in the results section and earn some CPD credits for their supervisory work [29]. The more skills the doctor masters the more completed EPAs they can have, and these can be recognised for CPD, or even in a notching scale for career advancement as an additional incentive [4].

### CPD relevant to the workplace (quality improvement and reflective diaries)

QI-CPD is a type of CPD that incorporates quality improvement into the assessment of current practice. It is an active process that encourages practitioners to measure practice metrics, compare these to peer feedback or benchmarks (where possible) and develop relevant, measurable action plans to improve practice [24]. Some of the recognised barriers to its implementation include lack of adequate training among healthcare practitioners, time constraints, and lack of incentives [24]. It is a viable method of CPD to use in the country as it aligns well with adult learning principles, as, according to Knowles (1985), adults want to be involved in planning and evaluation of their learning activities and prefer those learning activities that are most relevant to them and impact on their work [11]. In the case of QI-CPD practitioners identify their own learning needs and area of improvement and the learning process is directed at solving the identified problem with the aim of improving patient outcomes. By allowing quality improvement projects to count towards CPD credits, practitioners may feel motivated to participate in them [23]. As a recommendation, templates can be made available for easy completion. This would simplify the process of documenting the QI-CPD and of sending it forward for assessment.

The reflective diary is another useful CPD activity to consider in Eswatini. It is based on reflective practice, and an acknowledgement that reflection and feedback can be used as tools to develop not only knowledge but also skills [40, 41]. It is known that workplace learning can be enhanced through reflecting on experiences at work [24]. A key part in reflective practice is personalized and objective feedback, which must be provided for each reflective case sent for it to be effective [37]. Such feedback can utilise mentors, who can be specialists in the field and offer guidance and support or senior medical officers working in the facility [37]. Providing protected time for completion of reflective cases and making documenting these cases online/electronically has been shown to assist with improving compliance rates [37]. Using a validated framework such as Kirkpatrick’s learning evaluation (which is used for undergraduate learning) can be useful in evaluating the effectiveness of reflective activities [37].

### Limitations

The study findings cannot be generalised due to the small sample size and non-probability sampling of 10 general practitioners working in two Government hospitals in Eswatini. This means that potentially valuable contributions from those in private practice were not obtained. There is a risk of bias due to the dual role of first author as researcher and participant even though the authors did incorporate methods to promote rigor. Additionally, PAR as a research methodology was new for all our participants, as such there were a number who were not entirely comfortable with the approach even after the conscientization sessions, and this may have affected their participation. However, the points raised by participants are a useful indicator of the views and experiences of the participants on issues that affect practitioners undertaking CPD in Eswatini and are an important part of developing evidence-based advocacy for practitioners needs in as far as CPD is concerned.

## Conclusions

There are many options for CPD that can be used in Eswatini. The important consideration is involvement of local practitioners, ensuring use of adult learning principles, and using methods that allow evaluation and change to be implemented. It is encouraging to note that some of the recommendations from the policy development stage are being implemented with the EMDC-CPD committee now in place and it has made a written commitment to engage positively with practitioners. Further dissemination of the findings is in the process. The EPAs, reflective diaries and QI-CPD described are some potential forms that a CPD model can take as each meet most of the criteria for a suitable model. The study used a small group and focused on a few areas of CPD, many other aspects could be added to those suggested. Therefore, more research will still be needed to evaluate each of these in greater detail and their acceptability to the practitioners. Evaluation of CPD activities being currently provided in the country is also requires further study.

## Data Availability

The datasets used and/or analysed during the current study are available from the corresponding author on reasonable request.

## Abbreviations

CPD: Continuing professional development
PAR: Participatory action research
QI-CPD: Quality-Improvement– Continuing professional development
EMDC: Eswatini Medical and Dental Council
CIG: Cooperative inquiry group
AMCOA: Association of Medical Councils of Africa
EPA: Entrustable Professional Activities
